# Contributions of age of start, cognitive abilities and practice to musical task performance in childhood

**DOI:** 10.1371/journal.pone.0216119

**Published:** 2019-04-25

**Authors:** Kierla Ireland, Thanya A. Iyer, Virginia B. Penhune

**Affiliations:** 1 Laboratory for Motor Learning and Neural Plasticity, Concordia University, Montreal, Quebec, Canada; 2 International Laboratory for Brain, Music, and Sound Research (BRAMS), Montreal, Quebec, Canada; University of Zurich, SWITZERLAND

## Abstract

Studies with adult musicians show that beginning lessons before age seven is associated with better performance on musical tasks and enhancement in auditory and motor brain regions. It is hypothesized that early training interacts with periods of heightened neural development to promote greater plasticity and better learning and performance later in life. However, we do not know whether such effects can be observed in childhood. Moreover, we do not know the degree to which such effects are related to training, or whether early training has different effects on particular musical skills depending on their cognitive, perceptual or motor requirements. To address these questions, we compared groups of child musicians who had started lessons earlier or later on age-normed tests of rhythm synchronization and melody discrimination. We also matched for age, years of experience, working memory and global cognitive ability. Results showed that children who started early performed better on simple melody discrimination and that scores on this task were predicted by both age of start (AoS) and cognitive ability. There was no effect of AoS for the more complex rhythm or transposed melody tasks, but these scores were significantly predicted by working memory ability, and for transposed melodies, by hours of weekly practice. These findings provide the first evidence that earlier AoS for music training in childhood results in enhancement of specific musical skills. Integrating these results with those for adult musicians, we hypothesize that early training has an immediate impact on simple melody discrimination skills that develop early, while more complex abilities, like synchronization and transposition require both further maturation and additional training.

## Introduction

Studies in adults show that musicians who begin training before age seven show enhancements in behaviour and brain structure compared with those who begin later [1–6]. Based on this evidence, it is hypothesized that training during specific periods of brain maturation in childhood lead to greater plasticity and thus better learning and performance in the long term. However, all previous studies demonstrating the impact of early training are in adults with more than 10 years of experience; thus we do not know whether early training has immediate effects in childhood, or whether those effects require additional maturation and/or long-term practice to develop. Further, we do not know whether early training has different effects on specific musical skills depending on their cognitive, perceptual or motor requirements. Therefore, in this study we compared performance on tests of musical ability in groups of children who began lessons earlier or later but who were matched for years of experience and other relevant training and cognitive factors. In addition to standard matched-group comparisons we also used regression to assess the differential contribution of cognitive measures and training.

The first study to suggest that the age of start (AoS) of musical training might modulate brain plasticity compared the surface area of the corpus callosum in musicians and non-musicians [5]. They found that overall the anterior corpus callosum was larger in musicians, but that this effect was greater for those who began training before age seven. No specific rationale for this cut-off was given, either based on the trajectories of brain maturation or music training. Most importantly, there was no control for the normally high correlation between AoS and years of experience, with earlier AoS related to greater experience. Subsequently, a series of studies from our lab and others have examined the impact of early training on behavior and the brain using samples of early-trained (< 7; ET) and late-trained (>7; LT) musicians matched for years of experience, years of formal training, and hours of current practice. Behaviourally, ET musicians have been found to have more accurate performance on complex sensorimotor synchronization tasks than LT musicians, using both visual-motor and auditory-motor paradigms [2,3,6,7]. In the visual-motor domain, ET musicians outperformed LT musicians on a timed motor sequence task (TMST) for which they were trained to reproduce sequences of visually-presented ‘rhythms’ on a piano keyboard. The advantage for ET musicians was observed in training periods as short as two days [6] or five days [7]. In another sample, ET musicians had more precise timing than LT musicians during a task that required reading and playing scales from sheet music [8]. In the auditory-motor domain, ET musicians outperformed LT musicians on a rhythm synchronization task (RST) in which they listened, and then tapped along to each note of rhythms that varied in metrical complexity [3]. This finding was replicated in a second sample of ET and LT musicians with the same complex rhythmic task [2]. By contrast, in another sample of ET and LT musicians, no differences were found for a simple synchronization and continuation task [1]. Taken together, these results suggest that early training has greater long-term effects on more complex rhythmic tasks.

Longitudinal and quasi-experimental studies provide strong evidence that music training in childhood can produce changes in brain structure above and beyond normal maturation, and that these changes often correspond to improvements on musical tasks. Importantly, in these studies all children were found to be equivalent prior to music training in terms of brain structure, SES and cognitive abilities. In one study, six-year-old children were given 15 months of private keyboard lessons, and compared to a control group with only school-based music classes [9]. Those with private lessons showed enhancements in motor regions which were correlated with improvements on a melody and rhythm discrimination task. They also showed enhanced white-matter connectivity which correlated with improvements on a fine-motor sequencing task. Another group which followed children aged 6–18 for two years found a positive association between musical training and the rate of cortical thickness maturation in motor regions [10]. Very recently, six-year-old children were assigned to group music training following the El Sistema model, team sports training, or no systemic training [11]. After one year, children in the music group showed better performance on a task in which they synchronized drumming patterns with an adult [12]. After two years, children in the music group showed enhanced connectivity in the corpus callosum and better tonal discrimination compared to the two control groups [13]. Electrophysiological evidence suggests that changes in young children’s neural processing of sound occur after as little as one to three years of lessons. For example, auditory-evoked potentials were larger in amplitude in four- to five-year-old children after one year of music lessons [14]. In another study, responses to violin tones were heightened in children aged 4–6 after one year of Suzuki music lessons when compared to children without musical training [15]. Finally, children with and without musical training were assessed every two years from age 7–13. The auditory-evoked responses of children with musical training grew larger in amplitude with time, suggesting enhanced auditory processing above and beyond normal development [16].

As described above, no specific rationale has been developed for the age of seven cut-off used in previous work. One study from our lab attempted to validate this cut-off by examining the relationship between AoS and rhythm synchronization performance for different age cut-offs (6, 7, 8 and 9) in a large sample of adult ET and LT musicians [17]. For all cut-offs, AoS was more highly correlated with performance in the ET than the LT groups, with a significant difference for the age seven cut-off. These findings support the use of age seven as a boundary for early training, but they also indicate that it is not a hard cut-off.

Taken together, these findings indicate training may enhance the developmental trajectory for musical skills through interaction with normal maturation and plasticity in auditory and motor regions of the brain. Longitudinal studies with children show that one to three years of music lessons in childhood can lead to improvements in synchronization and pitch discrimination which are related to changes in the underlying neural substrates. Cross-sectional studies with adults show that, even when controlling for lifetime musical training, having started before age seven was associated with better sensorimotor musical abilities later in life. A non-linear relationship has been proposed between AoS and task performance, with better performance associated with early AoS up to age nine [17]. Therefore, the purpose of this study was to investigate the the relative contributions of AoS, music lessons, and music practice to musical task performance in childhood. A large sample of children aged 6–14 with music training were tested on Melody Discrimination and Rhythm Synchronization tasks developed in our lab [18,19]. Demographic and training information as well as measures of cognitive abilities were also collected. Based on previous studies in adults, we first examined group differences between ET and LT child musicians at a range of AoS cut-offs (5, 6 and 7) using age-equivalent z-scores. These groups were matched for years of lessons, cognitive and demographic variables. In a second step we assessed the relationship between AoS and performance between groups across these cut-offs. Finally, we used hierarchical regression to assess the individual contributions of AoS, cognitive and training variables to task performance.

## Materials and methods

### Participants

We tested 130 child musicians (age range: 6.50–14.08 years) from music day camps in Montréal, Ottawa, and Waterloo, Canada. We operationalized the term “musician” as a child fulfilling the following criteria: a) having at least 2.5 consecutive years of weekly, one-on-one music lessons on the same instrument (*M* = 5.06 years, *SD* = 1.58, range 2.58–10.00); (b) attending music lessons at time of recruitment; and (c) practicing music at least half an hour per week outside of lessons and on the same instrument (*M* = 3.16 hours, *SD* = 2.49, range 0.50–14.00). Children were eligible if they answered ‘yes’ to having at least one private music lesson per week. We did not inquire about duration of private lessons, or about group lessons. Music practice could be structured (using a book or specific exercises) or unstructured (free playing). Practice-related and demographic data were collected from parents on a questionnaire adapted in our lab (Survey of Musical Interests; [20]). We estimated SES using maternal years of education (*M* = 17.54 years, *SD* = 2.44, range 12.00–22.00). Mothers reported their highest level of education on an ordinal scale, and we converted this to an approximate interval scale with the following estimates: high school = 12 years; college diploma = 14 years; baccalaureate degree = 16 years; master’s degree = 18 years; doctorate or medical professional degree = 22 years. Parents provided written consent and children provided verbal assent before participating. Children were given a gift card and a small toy as thanks for their participation. The study was approved by Concordia University’s Human Research Ethics Board.

### Musical tasks

**Fig 1:**
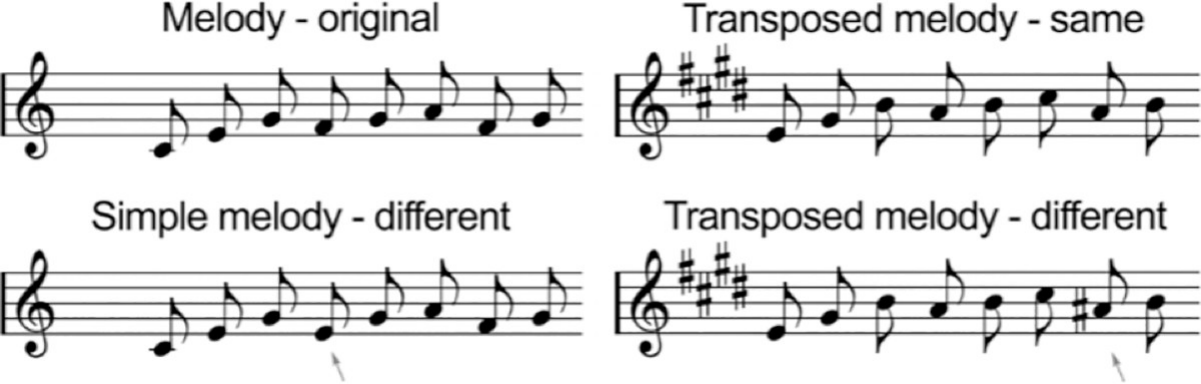
Examples of stimuli from the c-MDT Simple Melodies (L) and Transposed Melodies (R). Children listen to two melodies and decide whether the second was the same or different. Arrows represent the ‘different’ note. Figure adapted from [21].

**Fig 2:**
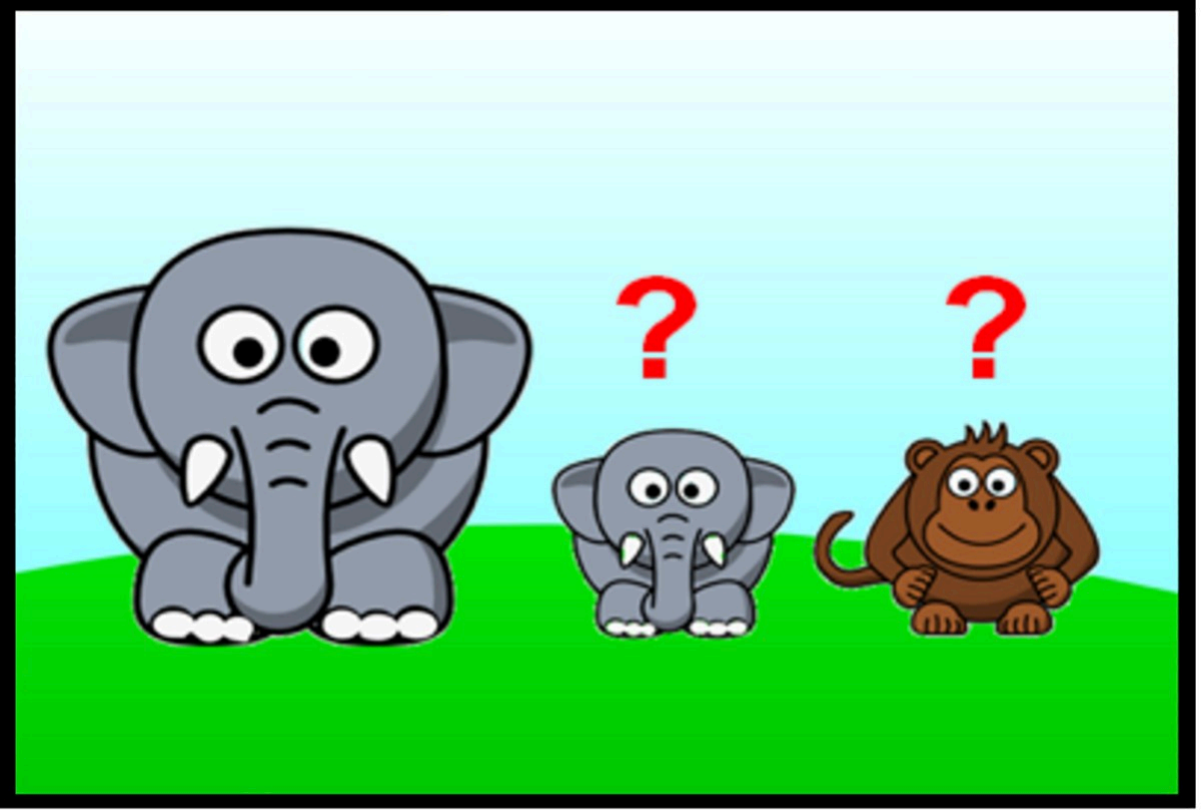
Graphical display of response probe for the c-MDT. Small elephant and monkey represent ‘same’ and ‘different’ response choices, respectively. Image is presented in full colour within the actual task.

For the children’s Melody Discrimination Task (c-MDT), children listen to two melodies of equal duration separated by a 1.2-second silence, and then indicate whether the second melody is the same or different (Figs 1 & 2). There are two conditions, Simple and Transposed, each with 20 trials (10 same and 10 different). In the Simple condition, both melodies are in the same key and in the “different” trials the pitch of a single note in the second melody is shifted up or down by up to five semitones. The child thus must compare individual pitches to detect the deviant note. In the Transposed condition, all the notes in the second melody are transposed upward by four semitones (a major third) and in the “different” trials a single note is shifted up or down by one semitone. Thus, since the melodic contour is preserved, the child must use relative pitch to perceive the deviant note. Melodies are composed of low-pass-filtered harmonic tones (320 ms) from the Western major scale (range: C4-E6). The 20 trials are presented in random order within conditions, but the order of conditions is always the same (Simple, Transposed) to preserve the storyline. Before starting each condition (Simple or Transposed), children are familiarized through four practice trials, two with feedback from the experimenter and two without feedback. The procedure for adapting the c-MDT from the adult version was recently published [18]. After all trials, the word ‘correct’ or ‘incorrect' is displayed for one second. During experimental trials, experimenters are seated so as not see children’s responses or feedback. Performance on the c-MDT is scored as the percentage of correct responses. The child's responses are scored as 0 (incorrect) or 1 (correct), generating a proportion which is then multiplied by 100. Given the evidence of transposition ability being anatomically distinct from other auditory discrimination abilities, Simple and Transposed melodies are always reported separately.

**Fig 3:**
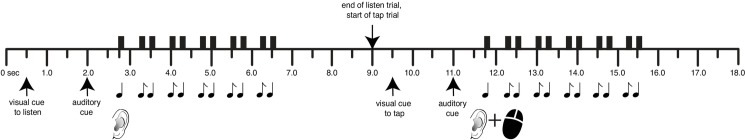
Examples of stimuli used in the c-RST. From top to bottom: low, medium, and high complexity rhythms. Low complexity rhythms (top) have the highest number of notes falling on the implied beat. Figure adapted from [19].

**Fig 4:**
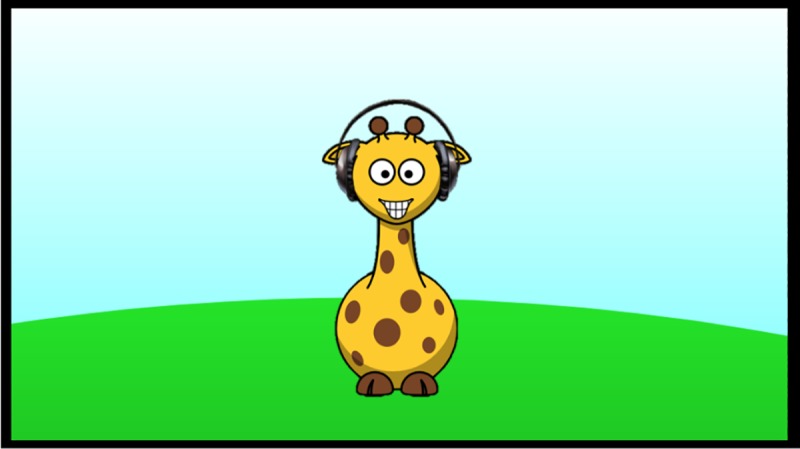
Graphical display for the c-RST. Giraffe’s headphones are highlighted during ‘listen’ phase, and hoof is highlighted during ‘listen + tap’ phase. Image is presented in full colour within the actual task.

For the children’s Rhythm Synchronization Task (c-RST), children listen and then try to tap along to each note of a rhythm while it plays, using the index finger on a computer mouse (Figs 3 & 4). Rhythms consist of 11 woodblock notes spanning an interval of 4 to 6 seconds. The c-RST has two rhythms at each of three levels of complexity (low, medium, high), for a total of six rhythms, which are presented in a counterbalanced order. Low complexity rhythms are repetitive and have a strong beat, whereas high complexity includes syncopated rhythms, which do not emphasize the beat. In a larger sample of children with and without musical training, ITI synchrony decreased consistently with increasing rhythmic complexity [18]. Thus, for this study, we used the average of all three complexity levels. A single trial of the c-RST consists of a ‘Listen’ phase and a ‘Tap in Synchrony’ phase, and each rhythm is presented for three consecutive trials (i.e., Listen-Tap; Listen-Tap; Listen-Tap). Before starting the test, children complete five practice trials at the low complexity level, with feedback from the experimenter. The rhythms used for the practice trials are not those used in the main task. Performance on the RST is measured in inter-tap interval (ITI) synchrony, or the child’s ability to reproduce the temporal structure of a rhythm. It is calculated as the ratio of the child’s response intervals (r) to the stimulus time intervals (t), with the following formula: Score = 1–abs(r–t)/t. This proportion is multiplied by 100 to generate a percentage, with scores closer to 100 indicating better synchrony.

### Cognitive tasks

We administered three subtests from the Wechsler Intelligence Scale for Children, fourth edition (WISC-IV; [22]): Digit Span (DS), Letter-Number Sequencing (LNS), and Matrix Reasoning (MR). Digit Span is a measure of immediate auditory memory, in which the child repeats strings of digits forward or backward. Letter-Number Sequencing (LNS) is a measure of auditory working memory, in which the child hears a string of letters and numbers and must repeat them back in numerical and alphabetical order, respectively. Together, DS and LNS comprise the Working Memory Index and are reported as such herein. Matrix Reasoning (MR) is a measure of nonverbal reasoning, and is considered to be a reliable estimate of general intellectual ability [23,24]. For this task, the child must identify the missing portion of an incomplete visual matrix from one of five response options. All subtests were administered according to standardized procedures. Raw scores were converted to scaled scores based on age-based norms.

### General procedure

Testing took place over a one-hour session. Participants were given short breaks between tasks to enhance motivation and prevent fatigue. Computer-based tasks were administered on a laptop computer running Presentation software (Neurobehavioral Systems, http://www.neurobs.com/). Task order was counterbalanced across participants. Auditory tasks were presented binaurally via Sony MDRZX100B headphones pre-adjusted to a comfortable sound level. Cognitive tasks were administered in the order in which they appear in the original WISC-IV battery.

## Results

### Examining group differences between ET and LT child musicians

We first examined average group differences between ET and LT children by creating three sub-samples based on AoS: age five (*N* = 110), age six (*N* = 96) and age seven (*N* = 52), with equal numbers of ET and LT musicians in each group. Children who had started prior to the cut-off were categorized as ET; those who had started at or after the cut-off were categorized as LT. To create matched samples at each AoS cutoff (age 5, 6 and 7) we first selected the group of ET children. Then, we matched an LT counterpart that resembled the ET child as closely as possible (+/- up to one-half of a standard deviation) on the following variables: years of music lessons, gender, Matrix Reasoning, Working Memory Index, Maternal Education and hours of weekly practice. For example, if an ET child had a WMI of 24, where *M* = 20 and *SD* = 6, we selected an LT counterpart with a WMI in the range of 21–27. Demographic, training-related, and cognitive characteristics of ET and LT musicians in the three sub-samples are presented in Tables 1–3.

**Table 1:** Matched demographic, practice-related and cognitive variables in early-trained (ET) and late-trained (LT) musicians; ET < 5 ≤ LT (*n* = 110).

Variable	ET (*n* = 55)	LT (*n* = 55)	*t* (108)	*p*	*g*
Maternal education (years)	17.94 (2.52)	17.40 (2.48)	1.03	0.30	0.20
Music lessons (years)	5.17 (1.45)	5.09 (1.23)	0.31	0.76	0.06
Weekly practice (hours)	3.28 (1.94)	2.95 (2.46)	0.78	0.44	0.15
Working Memory Index (scaled score)	23.76 (4.51)	22.51 (3.92)	1.55	0.12	0.30
Matrix Reasoning (scaled score)	12.47 (2.74)	12.31 (2.69)	0.31	0.76	0.06

**Table 2:** Matched demographic, practice-related and cognitive variables in early-trained (ET) and late-trained (LT) musicians; ET < 6 ≤ LT (*n* = 96).

Variable	ET (*n* = 48)	LT (*n* = 48)	*t* (94)	*p*	*g*
Maternal education (years)	17.97 (2.56)	17.08 (2.29)	1.79	.08	0.37
Music lessons (years)	4.65 (1.31)	4.59 (1.31)	0.22	.83	0.05
Weekly practice (hours)	2.80 (1.63)	2.14 (1.91)	1.81	.07	0.37
Working Memory Index (scaled score)	22.04 (3.04)	21.92 (4.69)	0.15	.88	0.03
Matrix Reasoning (scaled score)	11.98 (2.31)	12.13 (2.54)	0.30	.76	0.06

**Table 3:** Matched demographic, practice-related and cognitive variables in early-trained (ET) and late-trained (LT) musicians; ET < 7 ≤ LT (n = 52).

Variable	ET(*n* = 26)	LT(*n* = 26)	*t* (50)	*p*	*g*
Maternal education (years)	17.20 (2.27)	16.96 (2.60)	0.31	.76	0.09
Music lessons (years)	4.20 (1.08)	4.29 (1.08)	0.37	.71	0.10
Weekly practice (hours)	1.95 (1.75)	1.79 (1.50)	0.35	.72	0.10
Working Memory Index (scaled score)	21.69 (3.04)	21.92 (4.85)	0.20	.84	0.06
Matrix Reasoning (scaled score)	11.65 (2.41)	12.08 (2.37)	0.65	.52	0.18

For the c-RST we predicted that ET children would score higher than LT at an AoS cut-off of age seven, as in previous findings with adult musicians. There are no published studies of the c-MDT comparing ET and LT musicians; however, we previously found that Simple Melody scores were higher than Transposed Melodies at all ages [18]. Moreover, the neural correlates of transposition ability have been found to develop later in life [25]. Thus, we hypothesized that any AoS effects would be limited to Simple Melodies.

**Fig 5:**
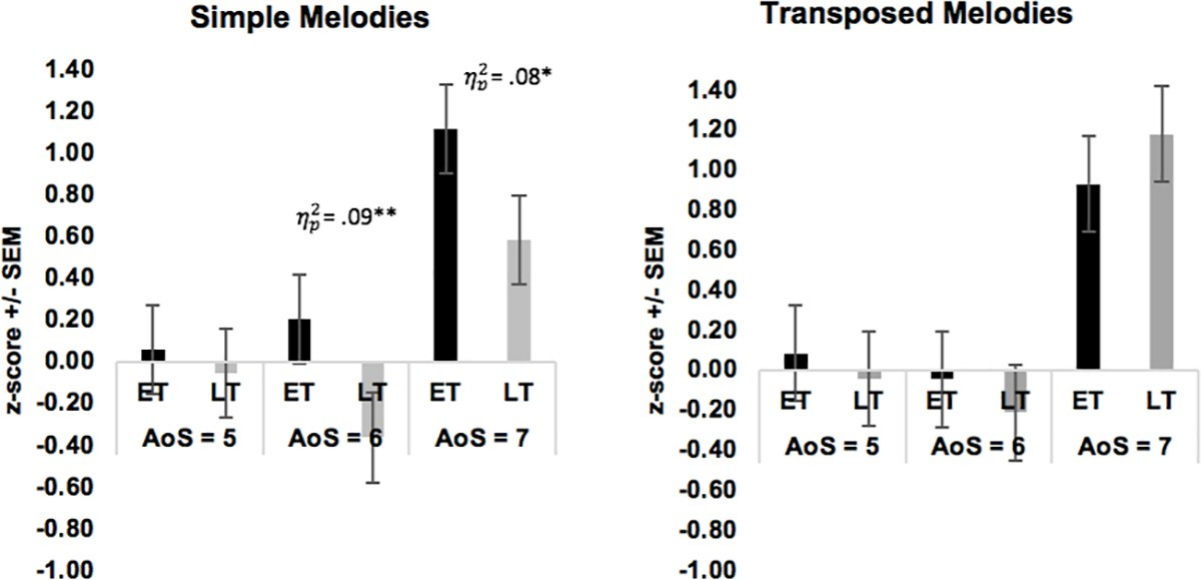
Results of one-way ANOVA for children’s Melody Discrimination Task (c-MDT). Bars represent early-trained (ET) and late-trained (LT) musicians at three age of start cut-offs. Performance is measured as age-based z-scores for Simple Melodies (L) and Transposed Melodies (R).

**Fig 6:**
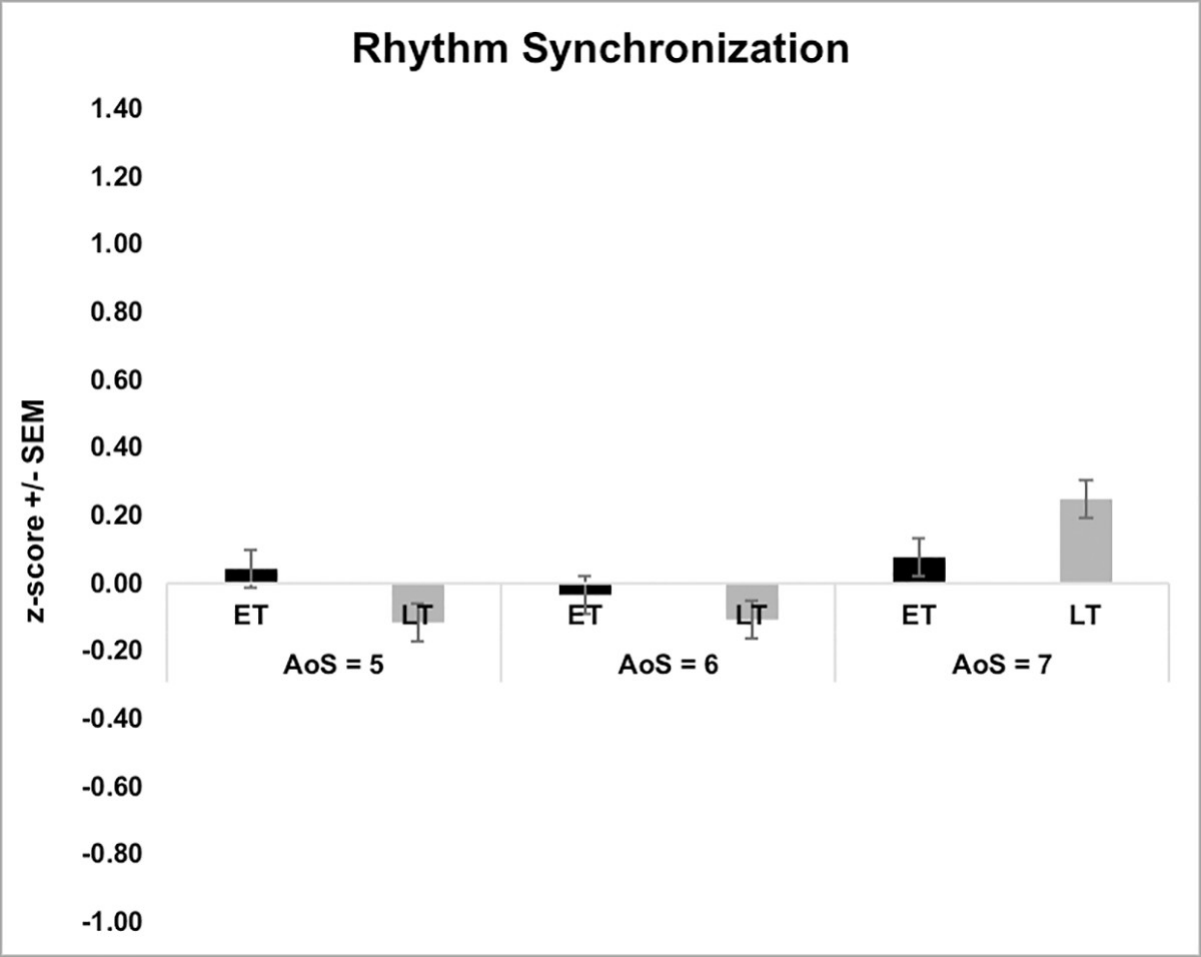
Results of one-way ANOVA for children’s Rhythm Synchronization Task (c-RST). Bars represent early-trained (ET) and late-trained (LT) musicians at three age of start cut-offs. Performance is measured as age-based z-scores.

We conducted a one-way analysis of variance (ANOVA) with group (ET or LT) as the two-level factor, at each AoS cut-off (5, 6, and 7) on the age-normed z-scores for each child for each outcome measure (c-MDT: Simple and Transposed Melodies; c-RST: ITI Synchrony; Figs 5 & 6). All assumptions for one-way ANOVA were met, including univariate normality, independence of observations, and homogeneity of variance [26]. There were no group differences at the first cut-off (ET < 5 **≤** LT) for any outcome (Simple Melodies: *F*(1, 108) = 0.39, *p* = .537, partial *η*^2^ = .004; Transposed Melodies: *F*(1, 108) = 0.41, *p* = .523, partial *η*^2^ = .004; ITI Synchrony: *F*(1, 108) = 0.39, *p* = .389, partial *η*^2^ = .007). . At the second cut-off (ET < 6 ≤ LT), ET musicians outperformed LT for Simple [*F*(1, 94) = 9.56, *p* = .003, partial *η*^2^ = .092] but not Transposed melody discrimination [*F*(1, 94) = 0.69, *p* = .407, partial *η*^2^ = .007]. Groups did not differ in ITI Synchrony [*F*(1, 94) = 0.13, *p* = .719, partial *η*^2^ = .001]. Similarly, at the oldest cut-off (ET < 7 ≤ LT), ET outperformed LT for Simple [*F*(1, 50) = 4.29, *p* = .043, partial *η*^2^ = .079] but not Transposed melody discrimination [*F*(1, 50) = 0.41, *p* = .524, partial *η*^2^ = .008] and there were no differences in ITI Synchrony [*F*(1, 50) = 0.61, *p* = .439, partial *η*^2^ = .012].

### Examining ET-LT cut-offs

To explore the validity of AoS cut-offs, we calculated the correlations between AoS and z-score for ET and LT groups for each task and at each cut-off. We then compared correlation coefficients between groups using Fisher’s z Transformation and z-test [27]. In addition, we calculated slopes using regression models and compared them using *t*-test analyses. In a previous study examining ET-LT cut-offs of 6, 7, 8, and 9 in adult musicians, younger age of start predicted better performance on the Rhythm Synchronization Task at all four cut-offs. Furthermore, correlations and slopes differed the most between ET and LT musicians when age seven was used to divide the groups [17]. This was taken as suggestive of an AoS effect for age seven. If such an effect is observable in childhood, we predict that younger age of start will correlate with better c-RST performance at all three cut-offs. Moreover, we expect that correlations and slopes will differ the most between ET and LT children at age seven for the c-RST.

Overall, there were no statistically significant differences in correlations or slopes at any cut-off. However, several patterns of correlations emerged in LT groups which corresponded to our hypotheses. For Simple Melodies, lower age of start was most strongly correlated with scores at the youngest cut-off, age five [*r*(53) = -.25, *p* = .069]. For Transposed Melodies, task performance was not correlated with AoS at any cut-off. For rhythm synchronization, lower AoS was most strongly correlated with scores at the oldest cut-off, age seven [*r*(24) = -.48, *p* = .013].

### Examining AoS as a predictor

Lastly, we conducted a hierarchical polynomial regression analysis [28] to examine the contributions of AoS, music lessons and practice, and cognitive abilities, as well as any non-linear relationships, to musical task performance. In contrast to the previous analyses in which we matched children in ET and LT groups, for this analysis we used the full dataset (*N* = 130), limiting the range of potential contributing factors while minimizing data loss. To do this, we converted raw values to standardized values, or used standard scores, for all predictors (age of start, years of lessons, hours of weekly practice, SES, WMI, MR). We removed all cases with an absolute value exceeding 2.50 SD on any of these predictors. This limited range resulted in the removal of 16 cases, for a total sample size of 114. As with the matched-subjects approach, we used age-based (z) scores for all musical tasks to control for differences due to maturation.

For each musical task, predictors besides AoS that were statistically significantly correlated with the outcome were added at step 1 as control variables (c-RST: WMI *r* = .28; c-MDT Simple: MR [*r* = .20]; c-MDT Transposed: WMI [*r* = .20], weekly practice [*r* = .25]). Our predictor of interest, the linear variable ‘age of start’ (AoS), was added at step 2. The power terms ‘AoS^2^’ and ‘AoS^3^’ were added at steps 3 and 4, to examine quadratic and cubic relations, respectively. To reduce multicollinearity with its power terms, we centered the variable AoS at its mean for each age group; this centered variable was used as the basis for all regression analyses [29].

For Simple Melodies, a linear regression model with only Matrix Reasoning accounted for 4.1% of the variance (*β* = .20, *t* = 1.89, adjusted *R*^2^ = .03, *p* = .031). Adding AoS contributed 3.2% independent variance to the model (*β* = -.18, *t* = -1.95, adjusted *R*^2^ = .06, *p* = .054). Power terms did not add any independent variance to the model.

For Transposed Melodies, a linear regression model with only Working Memory accounted for 4.0% of the variance (*β* = .20, adjusted *R*^2^ = .03, *p* = .034). Adding Weekly Practice Hours contributed 7.4% independent variance to the model (*β* = .27, adjusted *R*^2^ = .10, *p* = .003). Neither AoS nor any of its power terms accounted for independent variance to the model.

For Rhythm Synchronization, a linear regression model with only Working Memory accounted for 7.7% of the variance (*β* = .28, *t* = 3.06, adjusted *R*^2^ = .07, *p* = .003). Neither AoS nor any of its power terms accounted for additional variance to the model.

## Discussion

The results of this study showed that children who began training before age seven performed better on a simple melody discrimination task than those who started later, after being matched for musical training, demographic and cognitive variables. Further, both AoS and a measure of global intellectual function independently predicted scores on this task. There were no group differences or effects of AoS for the more complex rhythm synchronization and transposed melody discrimination tasks, but these were significantly predicted by working memory ability. Additionally, weekly practice was a strong independent predictor of transposed melody discrimination. These results provide clear evidence for the important contributions of maturational, training and cognitive factors in predicting musical task performance.

In the present sample, simple discrimination abilities were highest in those who had started music lessons before ages six and seven. Our finding of an AoS effect for simple pitch discrimination is supported by longitudinal studies showing that even short periods of music training during childhood can improve children’s discrimination of simple tones and melodies [13,30], neural processing of musical sounds and pitches [14–16,31], and accuracy in singing a simple melody [32]. This advantage for low-level pitch processing is likely a function of early maturation in the primary auditory cortex, in which there is a massive increase in the number of synapses and in myelination between ages one and five [33–36]. We and others have hypothesized that music training during periods of rapid maturational change may lead to greater brain plasticity that would promote enhanced learning both immediately and over the long term [6,37,38].

We also found that performance on simple pitch discrimination was related to global cognitive ability. There are several ways that cognitive and musical abilities might be related. On one hand, a global factor is posited to underlie the ability to approach and remain engaged with all cognitive tasks, resulting in positive correlations among these tasks, a phenomenon known as ‘the positive manifold’ [39]. This general ability would also support basic music-perceptual abilities which, like other cognitive processes, are hypothesized to be innate and normally distributed [40]. On the other hand, there is direct causal evidence that musical training during middle childhood, when compared to other types of training, can increase global cognitive ability [41]. Moreover, in a large longitudinal study of neuropsychological functioning across childhood, raw scores on tasks of global intellect increased sharply between ages 6–10 and reached adult levels by age 12–13 [42]. These maturational changes coincide with the time at which children in our sample are starting music lessons, and thus changes in cognitive abilities with maturation may also contribute to performance on music tasks. Altogether, our results provide evidence that music training before age seven results in specific gains in simple pitch discrimination, that are likely linked with developmental peaks in brain regions supporting basic auditory processing and with global cognitive development.

In contrast, we found no evidence that earlier start of training differentially contributed to rhythm synchronization ability. This is not consistent with results from studies with adult musicians showing that those who begin training before age seven outperform those who begin later on rhythm tasks [2,3,7,8]. However, our finding is consistent with the maturation of rhythmic abilities in childhood: beat perception is in place by infancy [43–45], but auditory-motor integration does not develop fully until mid- to late adolescence [46,47]. Moreover, rhythmic tapping tasks require basic fine-motor abilities that do not mature until late childhood [48,49]. Further, even with musical training, children’s rhythmic abilities take time to mature. For instance, children aged 6–8 improved on a tonal discrimination task after two years of music lessons, but rhythm discrimination did not appear to change [50]. Similarly, in children receiving music lessons from ages 7–13, there were improvements in the detection of pitch errors, but not timing errors, as measured with EEG [16,51]. To integrate the results of studies with child and adult musicians, we hypothesize that children must be older, have matured in terms of motor abilities, and have accrued substantial training to perform well on this task. This is supported by our previous findings using the same task with 7–13 year-old children showing continuing improvement with age, and that years of lessons contribute significantly to performance [18]. Finally, for rhythm synchronization we also found that children’s scores were significantly associated with working memory ability. This supports previous findings that scores on the task were correlated with measures of working memory in children [18] and adults [2,3].

For discrimination of transposed melodies, we also found no differences between matched ET and LT groups, and no effect of AoS. Similar to rhythm synchronization, there was a significant association between working memory and task performance. These findings demonstrate a strong, likely bidirectional relationship between musical training and working memory. On the one hand, playing music requires attending to and holding sequences of notes in mind, and applying the correct motor program to execute movements. These skills are supported by working memory which, like global cognitive function, develops most sharply between ages 6–10 and reaches adult levels by age 12–13 [42]. On the other hand, music practice directly enhances working memory through repetition of increasingly complex sensory-motor skills. Correspondingly, children with musical training have been found to have better performance on tasks of verbal and visuospatial working memory [52]. Thus, children with a better working memory capacity may be likely to engage in music training, and by doing so may enhance this skill.

We also found that hours of weekly practice, but not AoS or duration of musical training, significantly predicted transposed melody discrimination. This is consistent with findings from studies with adults that lifetime music practice accounted for more than two-thirds of the variance in performance on the same task [53]. This task is more difficult because it requires the participant to ignore contour, a highly salient auditory feature [54]. The only cue that differentiates the melodies is interval structure, or the change in pitch from one note to the next [55]. Children actively learn about interval structure through reading and repetition of musical scales during music practice. Thus, although non-musicians may understand implicitly, musicians’ explicit knowledge of interval structure enables them to perform much better on the transposed melody task. More than the other tasks in this study, transposed discrimination seems to require active engagement with music training, and with regular weekly practice specifically.

Our findings provide the first evidence in children that earlier start of music training results in better performance for simple melody discrimination, even when controlling for years of experience. This is likely a metaplastic effect where starting music training during a time of peak neurodevelopmental change produces better immediate and long-term learning. Performance for the more complex rhythm and transposition tasks did not show an effect of age of start and transposition ability was related to hours of practice. Performance for all music tasks was related to cognitive ability, indicating that cognitive skills likely both promote engagement in music and may be enhanced by training. Integrating these results with those for adult musicians, we hypothesize that early training has an immediate impact on skills like simple melody discrimination that develop early, while more complex abilities, like synchronization and transposition require both further maturation and additional training.

## References

[1] Baer LH, Park MT, Bailey JA, Chakravarty MM, Li KZ, Penhune VB. Regional cerebellar volumes are related to early musical training and finger tapping performance. Neuroimage. 2015 4 1;109:130–9. 10.1016/j.neuroimage.2014.12.076 25583606

[2] Bailey J, Penhune VB. A sensitive period for musical training: Contributions of age of onset and cognitive abilities. Ann N Y Acad Sci. 2012;1252(1):163–70. 22524355 10.1111/j.1749-6632.2011.06434.x

[3] Bailey JA, Penhune VB. Rhythm synchronization performance and auditory working memory in early- and late-trained musicians. Exp Brain Res. 2010;204(1):91–101. 10.1007/s00221-010-2299-y 20508918

[4] Bailey JA, Zatorre RJ, Penhune VB. Early musical training is linked to gray matter structure in the ventral premotor cortex and auditory-motor rhythm synchronization performance. J Cogn Neurosci. 2014;26(4):755–67. 10.1162/jocn_a_00527 24236696

[5] Schlaug G, Jancke L, Huang Y, Staiger JF, Steinmetz H. Increased corpus callosum size in musicians. Neuropsychologia. 1995 8;33(8):1047–55. 8524453 10.1016/0028-3932(95)00045-5

[6] Steele CJ, Bailey JA, Zatorre RJ, Penhune VB. Early musical training and white-matter plasticity in the corpus callosum: evidence for a sensitive period. J Neurosci. 2013;33(3):1282–90. 10.1523/JNEUROSCI.3578-12.2013 23325263 PMC6704889

[7] Watanabe D, Savion-Lemieux T, Penhune VB. The effect of early musical training on adult motor performance: evidence for a sensitive period in motor learning. Exp Brain Res. 2007;176(2):332–40. 10.1007/s00221-006-0619-z 16896980

[8] Vaquero L, Hartmann K, RipollÃ©s P, Rojo N, Sierpowska J, FranÃ§ois C, et al Structural neuroplasticity in expert pianists depends on the age of musical training onset. Vol. 126, NeuroImage. 2015. 10.1016/j.neuroimage.2015.11.008 26584868

[9] Hyde KL, Lerch J, Norton A, Forgeard M, Winner E, Evans AC, et al The effects of musical training on structural brain development: a longitudinal study. Ann N Y Acad Sci. 2009 7;1169:182–6. 10.1111/j.1749-6632.2009.04852.x 19673777

[10] Hudziak JJ, Albaugh MD, Ducharme S, Karama S, Spottswood M, Crehan E, et al Cortical Thickness Maturation and Duration of Music Training: Health-Promoting Activities Shape Brain Development. J Am Acad Child Adolesc Psychiatry [Internet]. 2014;53(11):1153–1161.e2. Available from: http://www.sciencedirect.com/science/article/pii/S0890856714005784 10.1016/j.jaac.2014.06.015 25440305 PMC4254594

[11] Habibi A, Damasio A, Ilari B, Sachs ME, Damasio H. Music training and child development: A review of recent findings from a longitudinal study. Ann N Y Acad Sci. 2018;1423(1):73–81. 10.1111/nyas.13606 29508399

[12] Ilari BS, Keller P, Damasio H, Habibi A. The Development of Musical Skills of Underprivileged Children Over the Course of 1 Year: A Study in the Context of an El Sistema-Inspired Program. Front Psychol [Internet]. 2016;7:62 Available from: 10.3389/fpsyg.2016.00062 26869964 PMC4735430

[13] Habibi A, Damasio A, Ilari B, Veiga R, Joshi AA, Leahy RM, et al Childhood Music Training Induces Change in Micro and Macroscopic Brain Structure: Results from a Longitudinal Study. Cereb Cortex [Internet]. 2017 11 8;1–12. Available from: 10.1093/cercor/bhw362 29126181

[14] Shahin A, Roberts LE, Trainor LJ. Enhancement of auditory cortical development by musical experience in children. Neuroreport. 2004;15(12):1917–21. 15305137 10.1097/00001756-200408260-00017

[15] Fujioka T, Ross B, Kakigi R, Pantev C, Trainor LJ. One year of musical training affects development of auditory cortical-evoked fields in young children. Brain. 2006;129(10):2593–608. 16959812 10.1093/brain/awl247

[16] Putkinen V, Tervaniemi M, Saarikivi K, Ojala P, Huotilainen M. Enhanced development of auditory change detection in musically trained school-aged children: A longitudinal event-related potential study. Dev Sci. 2013;17(2):282–97. 10.1111/desc.12109 24283257

[17] Bailey J, Penhune V. The relationship between the age of onset of musical training and rhythm synchronization performance: validation of sensitive period effects. Front Neurosci [Internet]. 2013;7:227 Available from: 10.3389/fnins.2013.00227 24348323 PMC3843222

[18] Ireland K, Parker A, Foster N, Penhune V. Rhythm and Melody Tasks for School-Aged Children With and Without Musical Training: Age-Equivalent Scores and Reliability [Internet]. Vol. 9, Frontiers in Psychology. 2018 p. 426 Available from: 10.3389/fpsyg.2018.00426 29674984 PMC5895917

[19] Tryfon A, Foster NE, Ouimet T, Doyle-Thomas K, Anagnostou E, Sharda M, et al Auditory-motor rhythm synchronization in children with autism spectrum disorder. Res Autism Spectr Disord. 2017;35:51–61.

[20] Desrochers A, Comeau G, Jardaneh N, Green-Demers I. L’élaboration d’une échelle pour mesurer la motivation chez les jeunes élèves en piano. Rev Rech en éducation Music. 2006;24:13–33.

[21] Karpati FJ, Giacosa C, Foster NE V, Penhune VB, Hyde KL. Sensorimotor integration is enhanced in dancers and musicians. Exp Brain Res. 2016;234(3):893–903. 10.1007/s00221-015-4524-1 26670906

[22] Wechsler D. WISC-IV technical and interpretive manual. San Antonio, TX: Psychological Corporation; 2003.

[23] Brody N. Intelligence. 2nd ed San Diego: Academic Press; 1992.

[24] Raven J., Raven J. C., and Court JH. Manual for Raven’s Progressive Matrices and Vocablary Scales Oxford, United Kingdom: Oxford Psychologists Press; 1998.

[25] Sutherland ME, Paus T, Zatorre RJ. Neuroanatomical correlates of musical transposition in adolescents: A longitudinal approach. Front Syst Neurosci. 2013;7. 10.3389/fnsys.2013.00113 PMC3865771 24381543

[26] Kline RB. Beyond significance testing: Reforming data analysis methods in behavioral research. Beyond significance testing: Reforming data analysis methods in behavioral research Washington, DC, US: American Psychological Association; 2004. xii, 325-xii, 325.

[27] Meng X, Rosenthal R, Rubin DB. Comparing correlated correlation coefficients. Psychol Bull. 1992;111(1):172–5.

[28] Cohen J, Cohen P, West SG, Aiken LS. Applied multiple regression for the behavioral sciences. Laurence Erlbaum, Hillsdale, NJ 1983;

[29] Kline RB. Principles and Practice of Structural Equation Modeling. 3rd ed. New York; 2011.

[30] Hyde KL, Lerch J, Norton A, Forgeard M, Winner E, Evans AC, et al Musical Training Shapes Structural Brain Development. J Neurosci. 2009;29(10):3019–25. 10.1523/JNEUROSCI.5118-08.2009 19279238 PMC2996392

[31] Besson M, Schön D, Moreno S, Santos A, Magne C. Influence of musical expertise and musical training on pitch processing in music and language. Restor Neurol Neurosci. 2007;25(3–4):399–410. 17943015

[32] Hutchins S. Early Childhood Music Training and Associated Improvements in Music and Language Abilities. Music Percept An Interdiscip J [Internet]. 2018;35(5):579–93. Available from: http://mp.ucpress.edu/lookup/doi/10.1525/mp.2018.35.5.579

[33] Kral A, Eggermont JJ. What’s to lose and what’s to learn: Development under auditory deprivation, cochlear implants and limits of cortical plasticity. Brain Res Rev [Internet]. 2007;56(1):259–69. Available from: http://www.sciencedirect.com/science/article/pii/S0165017307001877 10.1016/j.brainresrev.2007.07.021 17950463

[34] Moore JK. Maturation of human auditory cortex: implications for speech perception. Ann Otol Rhinol Laryngol. 2002;111(5_suppl):7–10. 10.1177/00034894021110s502 12018354

[35] Moore JK, Guan Y-L. Cytoarchitectural and axonal maturation in human auditory cortex. J Assoc Res Otolaryngol. 2001;2(4):297–311. 10.1007/s101620010052 11833605 PMC3201070

[36] Moore JK, Linthicum FH Jr. The human auditory system: a timeline of development. Int J Audiol. 2007;46(9):460–78. 10.1080/14992020701383019 17828663

[37] Altenmueller E, Furuya S. Brain Plasticity and the Concept of Metaplasticity in Skilled Musicians In: Laczko J, Latash ML, editors. Progress in Motor Control: Theories and Translations [Internet]. Cham: Springer International Publishing; 2016 p. 197–208. Available from: 10.1007/978-3-319-47313-0_11 28035567

[38] Herholz SC, Zatorre RJ. Musical Training as a Framework for Brain Plasticity: Behavior, Function, and Structure. Neuron [Internet]. 2012;76(3):486–502. Available from: 10.1016/j.neuron.2012.10.011 23141061

[39] Kovacs K, Conway ARA. Process Overlap Theory: A Unified Account of the General Factor of Intelligence. Psychol Inq [Internet]. 2016;27(3):151–77. Available from: 10.1080/1047840X.2016.1153946

[40] Glenn Schellenberg E, Winner E. Music Training and Nonmusical Abilities: Introduction. Music Percept An Interdiscip J [Internet]. 2011;29(2):129–32. Available from: http://www.jstor.org/stable/10.1525/mp.2011.29.2.129

[41] Schellenberg EG. Music lessons enhance IQ. Psychol Sci. 2004;15(8):511–4. 10.1111/j.0956-7976.2004.00711.x 15270994

[42] Waber DP, Moor C De, Peter W. Forbes CRA, Botteron KN, Leonard G, Milovan D, et al The NIH MRI study of normal brain development: performance of a population based sample of healthy children aged 6 to 18 years on a neuropsychological battery. J Int Neuropsychol Soc. 2007;13(5):729–46. 10.1017/S1355617707070841 17511896

[43] Hannon EE, Nave‐Blodgett JE, Nave KM. The Developmental Origins of the Perception and Production of Musical Rhythm. Child Dev Perspect. 2018;

[44] Van Noorden L, De Bruyn L. The development of synchronization skills of children 3 to 11 years old. In: Proceedings of ESCOM—7th Triennial Conference of the European Society for the Cognitive Sciences of Music Jyväskylä, Finland: University of Jyväskylä. 2009.

[45] Winkler I, Háden GP, Ladinig O, Sziller I, Honing H. Newborn infants detect the beat in music. Proc Natl Acad Sci. 2009;106(7):2468–71. 10.1073/pnas.0809035106 19171894 PMC2631079

[46] Drewing K, Aschersleben G, Li S-C. Sensorimotor synchronization across the life span. Int J Behav Dev [Internet]. 2006 5 1;30(3):280–7. Available from: 10.1177/0165025406066764

[47] Savion-Lemieux T, Bailey JA, Penhune VB. Developmental contributions to motor sequence learning. Exp brain Res. 2009 5;195(2):293–306. 10.1007/s00221-009-1786-5 19363605

[48] Gerber RJ, Wilks T, Erdie-lalena C, Gerber RJ, Wilks T. Developmental Milestones: Motor Development. 2010; 10.1542/pir.31-7-267 20595440

[49] Monier F, Droit-Volet S. Development of sensorimotor synchronization abilities: Motor and cognitive components. Child Neuropsychol [Internet]. 2019 2 4;1–20. Available from: 10.1080/09297049.2019.1569607 30714466

[50] Habibi A, Cahn BR, Damasio A, Damasio H. Neural correlates of accelerated auditory processing in children engaged in music training. Dev Cogn Neurosci [Internet]. 2016;21:1–14. Available from: 10.1016/j.dcn.2016.04.003 27490304 PMC6987702

[51] Meyer M, Elmer S, Ringli M, Oechslin MS, Baumann S, Jancke L. Long-term exposure to music enhances the sensitivity of the auditory system in children. Eur J Neurosci. 2011;34(5):755–65. 10.1111/j.1460-9568.2011.07795.x 21848923

[52] Bergman Nutley S, Darki F, Klingberg T. Music practice is associated with development of working memory during childhood and adolescence. Front Hum Neurosci. 2014;7:926 10.3389/fnhum.2013.00926 24431997 PMC3882720

[53] Foster NE V, Zatorre RJ. Cortical structure predicts success in performing musical transformation judgments. Neuroimage. 2010;53(1):26–36. 10.1016/j.neuroimage.2010.06.042 20600982

[54] Dowling WJ, Fujitani DS. Contour, Interval, and Pitch Recognition in Memory for Melodies. J Acoust Soc Am [Internet]. 1971;49(2B):524–31. Available from: http://asa.scitation.org/doi/10.1121/1.1912382 10.1121/1.1912382 5541747

[55] McDermott JH, Keebler M V, Micheyl C, Oxenham AJ. Musical intervals and relative pitch: Frequency resolution, not interval resolution, is special. J Acoust Soc Am. 2010;128(4):1943–51. 10.1121/1.3478785 20968366 PMC2981111

